# Barriers, Limitations, and Experiences with Clinical Trials—Treatment in Rare Diseases with Prader–Willi Syndrome as an Example

**DOI:** 10.3390/genes16121436

**Published:** 2025-12-01

**Authors:** Merlin G. Butler, Spencer Silvey, Harold J. P. van Bosse

**Affiliations:** Departments of Psychiatry & Behavioral Sciences and Pediatrics, University of Kansas Medical Center, Kansas City, KS 66160, USA

**Keywords:** barriers and limitations, clinical trials and experiences, rare diseases, Prader-Willi syndrome, *KCNJ11* gene and protein interactions, functions, and mechanisms, diazoxide choline

## Abstract

**Background/Objectives**: Developing and implementing clinical trials for rare diseases is complicated by the incomplete understanding of the varied genotype and subsequent phenotypic differences of a condition, particularly when low numbers of subjects are enrolled in a study. Moreover, a small-scale clinical study may indicate a positive outcome but have too small of a sampling population to adequately evaluate unwanted outcomes. Prader–Willi syndrome (PWS) is one such genetic disorder with varied subtypes and heterogeneity, where little progress has been made in treatment discoveries. Recently, the FDA approved diazoxide choline for treating key features of hyperphagia and obesity associated with PWS based on clinical trial experience. Diazoxide choline activates the ATP-sensitive potassium channel (KATP) of pancreatic beta cells, inhibiting the release of insulin. One of the subunits of KATP is the protein Kir6.2, the gene product of *KCNJ11*. **Methods**: Web-based programs and datasets were used to study the gene and protein functional enrichments of Kir6.2 and *KCNJ11*, including shared gene and/or protein–protein interactions, and biological processes and functions. **Results**: Four essential domains of related functions were identified: (1) apoptosis, protein degradation, and inflammation; (2) the coupling of G proteins needed for KATP channel activation; (3) glucose metabolism and control; and (4) the maintenance of intracellular ionic homeostasis. **Conclusions**: Cellular metabolism in the pancreas is linked to membrane excitability by KATP, which regulates insulin production, energy production and storage, appetite regulation, and fatty acid synthesis. As such, diazoxide choline may influence several biological systems beyond pancreatic and metabolic functions.

## 1. Introduction

### 1.1. Clinical Barriers to Treatment Discovery in Rare Diseases

Rare diseases are defined as affecting fewer than 200,000 individuals in the United States or no more than 1 in 2000 persons in any World Health Organization-defined region of the world [1,2]. Providing medical care and developing novel treatment options for patients with rare diseases in general are complicated by a paucity of awareness and natural history information. Advances in genomic technology and diagnostic testing have led to important breakthroughs but have not been utilized consistently worldwide. Delays in an individual’s diagnosis or its confirmation may impede timely selection for treatment or inclusion in clinical trials. The rarity of a condition further impacts efforts to enroll sufficient subject numbers in clinical trials or research studies. A fundamental understanding of biology related to the specific genetic defects of a rare disorder is obviously crucial in the selection of therapeutic agents in clinical trials and is often lacking in rare diseases. Issues related to pharmacogenetics and pharmacodynamics may arise, influencing the appropriate route of drug administration, dosage, and timing to maintain blood levels. Extended use may lead to drug resistance or altered outcomes. Other potentially unrecognized barriers may develop in clinical trials administered across multiple sites, locations and countries with different language and medical care practices. The recruitment of participants with genetic heterogeneity and from different age groups may introduce complicating factors difficult to fully address in a clinical trial. These are among the many as yet not fully appreciated issues that may impact clinical trial success in rare diseases.

We will focus on the clinical and genetic findings in Prader–Willi syndrome (PWS, OMIM#176270) with clinical trial experiences that led to discovery of the first FDA approved drug to treat hyperphagia and obesity in this rare genetic disorder. We will describe the gene and protein interactions, functional enrichment and mechanisms using web-based programs and databases that may relate to actions of the newly approved drug.

### 1.2. Clinical Findings in Prader–Willi Syndrome

Prader–Willi syndrome (PWS) is a rare disease that occurs in about one in 20,000 births [3,4], with an incidence of 200 babies per year in the USA [5] and a worldwide prevalence of 400,000 affected [6]. It is a rare complex genomic imprinting disorder, considered the most common genetic cause of marked obesity in humans [7]. It is characterized by early onset failure to thrive with severe infantile hypotonia, a poor suck, decreased saliva, and swallowing difficulties. Hypogonadism and hypogenitalism are present along with small hands and feet. Growth hormone and other hormone deficiencies are associated with decreased muscle mass, a lower metabolic rate, and decreased physical activity [4,8,9,10]. Cranio-facial dysmorphic features such as almond-shaped eyes, a triangular mouth and narrow forehead are noted. Mild learning and behavioral problems manifest including self-injury, compulsions, stubbornness and outbursts often related to food insecurity [7,9,10,11]. Food seeking/preoccupation and related behaviors, accompanied by a lack of satiety, begin in early childhood and are further complicated by the reduced metabolic needs of persons with PWS; the caloric needs of those with PWS are 30–50% less than their unaffected peers, and if their diet is not externally controlled, they will develop morbid obesity [3,10,12]. Hyperphagia coupled with the lack or inability to vomit can also be life-threatening.

The average age of death in PWS is reported at 30 years in both males and females, although this may be improving with contemporary treatments including weight restriction and growth hormone [13]. The most common cause of death in PWS is respiratory failure followed by cardiovascular and gastrointestinal issues, with choking deaths reported more often in childhood [13]. Despite the need to improve both the quality and span of life of those with PWS, the rarity of the condition complicates obtaining funding for research and clinical trials, hampering therapy development and drug discovery worldwide.

### 1.3. Genetics of Prader–Willi Syndrome

PWS results from the loss of expression of imprinted genes in the paternally derived 15q11–q13 region (e.g., [3,4]). Several PWS molecular genetic classes are recognized; the most common (about 60%) are the typical paternal 15q11–q13 deletion subtypes, followed by maternal disomy chromosome 15 subclasses (35%) where both chromosome 15s are inherited from the mother. The remaining 5% of patients have either a defect of the imprinting center that controls the genes on chromosome 15, or other chromosome 15 rearrangements [14,15]. The recurrence risk is low (<1%) for those with the typical 15q11–q13 deletions or maternal disomy 15, but families with offspring having microdeletions of the imprinting center may have a 50% risk of future affected children.

Deletions of the 15q11–q13 region have two typical subtypes, a larger and a smaller deletion, as well as rare atypically sized deletions. PWS deletions are defined by breakpoints; breakpoint 3 (BP3) is located at the distal end of the 15q11–q13 region and is common to both deletion subtypes. There are two proximal breakpoints, with BP1 close to the centromere, and BP2 at about 500 kilobases distal to BP1. The larger deletion, Type I, spans from BP1 to BP3, and the smaller Type II deletion extends from BP2 to BP3 distally. The rare atypical proximal 15q deletions can be larger or smaller than the typical deletions. Those with the typical deletion forms of PWS are more clinically affected than those with disomy and are prone to more self-injurious and compulsive behaviors and generally have lower cognition [16,17]. Clinical differences have been noted between the two deletion subtypes, with more severe clinical findings in those with the Type I deletion. The Type I deletions lack four additional proximal genes in comparison to the smaller Type II deletions. These four non-imprinted genes are *TUBGCP5*, *CYFIP1*, *NIBPA1*, and *NIPA2*, with the latter two genes coding for the transporters of magnesium and other cations, which may account for the disturbed magnesium levels in those with PWS having the Type I deletion [18]. A condition where only the four genes between breakpoints BP1 and BP2 are missing is known as Burnside–Butler syndrome [9,19,20].

There are three subclasses of maternal disomy 15: heterodisomy, total isodisomy, and segmental isodisomy. In all three maternal disomy 15 subclasses, both chromosome 15s are inherited from the mother and none from the father. The heterodisomy and segmental isodisomy subclasses represent the mother’s maternal and paternal chromosome 15 with or without cross-over events and result from errors in meiosis I. For total isodisomy 15, the two chromosome 15s from the mother result from errors in meiosis II and each of the chromosome 15s from the mother are identical (e.g., [3,4,9,15]). Those with maternal disomy 15 are at a greater risk of autistic features and psychosis during late adolescence or early adulthood with delayed diagnosis [3,4,9,11,21,22].

The rarer genetic classes of PWS include imprinting center defects with microdeletions or epimutations, and other chromosome 15 abnormalities, such as translocations or inversions [4,9,11,15,23,24]. Altogether, there are dozens of potentially altered imprinted and non-imprinted genes found in the 15q11–q13 region that determine the phenotypic and clinical variability, and severity of involvement, and may help individualize selection of options in treating those with PWS [3,4,11,24]. We have limited information on epigenetic-genomic effects in PWS but do know that often prescribed caloric restricted diets can affect both nutrients and vitamins when managing their proclivity towards obesity, interspersed with bouts of excessive caloric intake which may have a negative regulatory effect on gene control and protein production. This activity makes the biology of PWS even more variable and disturbed, impacting functions at the cellular level, such as important cations/electrolytes transport and regulation needed for normal and stable membrane ion channel function.

### 1.4. Clinical Trial Experiences in Prader–Willi Syndrome

Multiple approaches have been undertaken in an effort to discover therapies for managing hyperphagia and obesity in PWS, most with only limited success [3,4,25,26,27,28]. The use of diet restrictions and limiting access to food, increased physical activity and exercise, and growth hormone replacement have limited efficacy on appetite and weight control; successes occur at the level of the individual rather than the group. More intensive treatments, such as bariatric surgery, have inconclusive outcomes and are controversial in treating PWS [3,29,30].

#### 1.4.1. Non-Medication Interventions

Non-medication investigations and trials include transcranial direct-current stimulation (tDCS), a safe, painless method to modify neuronal and cognitive function in areas of the brain to assist in modulating food craving [31,32,33]. It has been applied to individuals with PWS from 16 to 65 years with success. The dorsolateral prefrontal cortex (DLPFC) is involved in the regulation and processing of food craving and hyperphagia. Active tDCS sessions in subjects with PWS targeting the DLPFC were associated with significant changes from baseline using the Three Factor Eating Questionnaire, both in the disinhibition component and the total score [33]. Furthermore, tDCS-treated patients trended towards a decreased response time on Go/NoGo task performance involving food and non-food stimuli images. The results further demonstrated the feasibility of examining neuromodulator effects on information processing in individuals with PWS and hold promise for potential use, but more research is needed.

Vagal nerve stimulation has been used in PWS to treat food-related behaviors, hyperphagia, and autonomic nervous system dysfunction (e.g., [34]). Other behavioral issues have been studied, including outbursts, physical aggression, and self-injury (e.g., [27,35,36]). There is growing evidence that vagal nerve stimulation may positively impact weight loss, food-related behaviors, circadian activity, and temper outbursts [37].

#### 1.4.2. Medication Interventions

Beloranib was one of the first pharmacologic interventions specifically developed to treat hyperphagia and obesity in those with PWS and studied in a clinical trial. It inhibits the methionine aminopeptidase 2 (MetAP2) enzyme by removing a methionine residue, which impacts fat metabolism and adipocyte size in animal models [38]. The clinical trial included individuals with PWS from 12 to 65 years of age and found a reduction in food intake, effects on adipose tissue, and weight loss in the participants. Unfortunately, the clinical trial and product development were discontinued following serious adverse events, including two deaths.

A different pharmacological approach targets circulating ghrelin levels or its bioavailability. Ghrelin is a neuropeptide produced by the stomach and stimulates appetite at the hypothalamus level [39]. Agents have been developed to alter either unacylated (inactive) ghrelin (UAG) or acylated (active) ghrelin (AG) levels [40]. Levoletide was formulated as an inactive ghrelin analogue to compete with active ghrelin in the brain of individuals with high ghrelin levels. In a clinical trial of individuals with PWS from 8 to 65 years, the drug was well tolerated, but without a significant change in hyperphagia [41]. There are preliminary efforts to design inhibitors of the enzymes that catalyze ghrelin (e.g., ghrelin O-acyltransferase) and thereby reduce production of AG and increase levels of UAG.

Oxytocin, a primitive neuropeptide hormone, plays a role in social interactions and skills, food intake, anxiety, energy expenditure, and body-weight regulation. Oxytocin binds to G protein-coupled receptors leading to secondary messengers that regulate appetite with a possible role in hyperphagia control. Decreased oxytocin-producing neurons in the brain suggest a possible role in regulating food intake and behavior [26]. Carbetocin, an oxytocin analogue, has undergone multiple clinical trials from infants to adults with PWS, but with equivocal outcomes as related to hyperphagia, food intake, and behavior [42,43,44,45,46,47,48].

Cannabinoid-1 receptor (CB1R) antagonists have been proposed as a treatment option for individuals with PWS. Theoretically, they would overstimulate the endocannabinoid system to reduce appetite, affecting body weight and secondarily, obesity-related metabolic disorders, without negatively impacting the central nervous system [49,50]. CB1R receptors are densely expressed in the hypothalamus and involve appetite control but are also found in other organs impacting energy utilization and storage, such as the liver, adrenal and pituitary glands, gonads, and adipose tissue, complicating target effects. Cannabidiol is a CB1R antagonist currently undergoing phase 2 clinical trials in pediatric patients with PWS [26].

Setmelanotide activates the melanocortin 4 receptors (MC4R) in the hypothalamus, resulting in an inhibition of food intake, and therefore has potential usefulness in treating PWS. A phase 2 clinical trial, though, showed no difference compared to placebo in those with PWS between 16 and 25 years [51,52]. However, it has met with success in other obesity-related genetic conditions such as Bardet–Biedl syndrome and hypothalamic obesity [3,4,25,53].

Another promising development is the pairing of two medications that have different effects. Tesofensine is a triple monoamine reuptake inhibitor, which inhibits reuptake of three key neurotransmitters in the brain, thereby increasing their availability. Tesofensine has been found effective in suppressing appetite, increasing satiety, and possibly increasing metabolism. Metoprolol is a beta-1 selective blocker, which can offset the hypertensive and tachycardic side effects of tesofensine. Tesomet is a combination of tesofensine and metoprolol, which demonstrated reductions in hyperphagia and body weight for adults and adolescents with PWS in phase 2 trials [54].

Glucagon-like peptide-1 (GLP-1) receptor agonists (GLP1RA) have garnered substantial attention due to their clinical efficacy in treating obesity in the general population. The medications mimic the actions of naturally occurring GLP-1, a hormone released in the gastrointestinal tract in response to eating. GLP-1 stimulates insulin release from the pancreas while lowering glucagon levels, both effects lower serum glucose levels. Additional effects of the GLP1RA medications are delayed gastric emptying with slowed small intestine mobility, and effects on the brain that help suppress appetite [55,56,57]. For this reason, the GLP1RAs are considered potentially helpful in PWS and in other obesity-related genetic disorders [58]. To date, the most studied GLP1RAs in PWS are liraglutide and exenatide and included patients with PWS with or without type 2 diabetes mellitus. Overall results show that most patients benefit from the treatment, but sometimes with limited results [57,58]. A large concern is the decreased gastric and gut motility related to these drugs, which could be potential risk factors in treating those with PWS who are at risk for gastroparesis.

ARD-101 is a new investigational oral drug entering phase 3 studies to treat hyperphagia and aggressive food-seeking behaviors in PWS. It is an agonist of the bitter taste receptor TAS2R, activating secretion of several gut peptide hormones including glucagon-like peptides-1 and -2 (GLP-1, GLP-2), which are involved in regulating eating behavior and metabolism [59]. ARD-101 also stimulates the secretion of cholecystokinin (CCK), which itself acts as a satiety signal via the gut–brain axis to control hunger. In PWS, it is thought that CCK release from the gut enteroendocrine I-cells in response to food is impaired leading to extreme hunger.

Other FDA-approved drugs that may have the potential to treat hyperphagia and decrease obesity in PWS include lorcaserin, naltrexone HCl/bupropion HCl, and phentermine/topiramate but more research with controlled trials is needed (e.g., [27]. These and other potential therapeutic agents are at different stages of development and use in treating PWS or other obesity-related disorders [60,61].

### 1.5. Diazoxide Choline Controlled Release (DCCR) (VYKAT XR)

The most successful clinical trial to date to treat hyperphagia in PWS is diazoxide choline controlled release, marketed as VYKAT XR [62,63]. In clinical trials of 127 participants with PWS, 4 years and older, diazoxide choline decreased hyperphagia, and improved Global Impression Scores and body composition [63], leading to FDA approval of VYKAT XR in April 2025, as the first drug specifically for hyperphagia in PWS. The studies did note several common side effects, including hirsutism/hypertrichosis (excessive hair growth), peripheral edema (swelling in the extremities), questionable muscle weakness and strength, and hyperglycemia (high blood sugar). These side effects were generally mild, and many resolved with continued treatment. Other studies that did not include patients with PWS reported pulmonary hypertension and heart failure [64].

**Figure 1:**
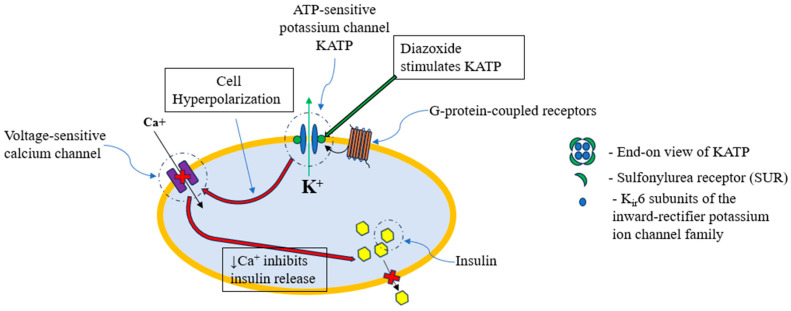
In beta cells of the pancreas, the ATP-sensitive potassium channel (KATP) is stimulated by diazoxide, causing an efflux of potassium ions. The efflux causes a hyperpolarization of the cell membrane, inhibiting calcium influx via the voltage-sensitive calcium channels. The decreased levels of intracellular calcium secondarily inhibit the release of insulin.

The drug plays a role in activating the ATP-sensitive potassium channel (KATP) that resides in cell membranes [65,66,67]. KATP channels are G protein-coupled receptors, found in many areas, including the beta cells of the pancreas. At rest, the KATP favors potassium ingress but when stimulated by G proteins, the KATP channel opens, allowing for an efflux of potassium ions out of the cell. The cation efflux causes a hyperpolarization of the cell membrane, which prevents voltage-dependent calcium channels from opening, inhibiting the influx of calcium into the cell. The influx of calcium ions is crucial for insulin release, and preventing their influx inhibits insulin secretion (e.g., [65]). The KATP is a hetero-octomeric structure with four copies of the sulfonylurea receptor (SUR) and four copies of the transmembrane potassium inward-rectifying (Kir6.2) channels [65,68,69,70] (Figure 1). In the pancreas, diazoxide choline binds the sulfonylurea receptor 1 (SUR1) subunit which opens the KATP channels on the beta cell membrane, leading to the inhibition of insulin release. In the brain, particularly the hypothalamus, diazoxide is believed to reduce the secretion of neuropeptides like NPY and AgRP, which are potent appetite stimulants, thereby helping to control hyperphagia [65].

The Kir6.2 protein is encoded by the *KCNJ11* gene (Potassium Inwardly-Rectifying Channel Subfamily J Member 11, OMIM *600937), and the SUR1 and SUR2 subunits are encoded by the *ABCC8* and *ABCC9* genes, respectively [65,67,68,71,72] (Figure 1). The Kir6.2 protein promotes the inward movement, or ingress, of potassium ions into the cell rather than outward movement, or egress. The voltage dependence is regulated by the concentration of extracellular potassium, where increased concentrations cause the voltage of the channel opening to shift more positively. The inward rectification is due to the preferential blockage of outward potassium currents by intra-cellular magnesium ions. This phenomenon can be reversed by removing magnesium or blocked by extracellular ions such as barium. Defects in the *KCNJ11* gene may contribute to autosomal dominant non-insulin-dependent diabetes mellitus type 2, transient neonatal diabetes mellitus type 3, and permanent neonatal diabetes mellitus [e.g., GeneCards (www.genecards.org); Online Inheritance in Man (www.omim.org) and STRING (www.string-db.org)] [65,66,67,71,72]. Given the complex interactions at play, factors that affect the functioning of KATP or its components may lead to unanticipated downstream effects but often not found in relatively small studies. The high costs and insufficient number of genetically confirmed participants with PWS or other rare disorders available for recruitment, and their genetic heterogeneity, make large-scale studies very challenging.

Diazoxide choline binds the SUR protein subunits of the KATP structure, with close interaction with Kir6.2 subunits, making *KCNJ11* a suitable proxy to study the gene–protein interrelationships associated with the diazoxide choline–KATP interactions. Our purpose was to study diazoxide choline’s predicted functions and mechanisms based on genetics and biology as they relate to PWS. Our aims were (1) to use searchable website-based genetic-protein programs and databases to identify associated or predicted protein–protein functional partners for the *KCNJ11* gene, including the identification of possible disease–gene associations; (2) to identify gene–gene functional interactions for gene binding and co-expression information; and (3) to categorize the findings based on their ontology through several computational biology formats.

## 2. Materials and Methods

Comprehensive searches were undertaken to utilize published literature and interactive web-based programs and databases, querying the terms *KCNJ11*, Kir6.2, Prader–Willi syndrome, and diazoxide choline in humans using PUBMED (https://pubmed.ncbi.nlm.nih.gov), Gene Cards (http://www.genecards.org), and OMIM (http://www.omim.org). We extensively utilized searchable publicly available web-based programs and databases. These programs were as follows: STRING (www.string-db.org) to study protein–protein interactions and molecular functions; BioGRID (https://theBioGRID.org) for protein–protein interactions; and PathwayCommons (www.PathwayCommons.org) for gene–gene interactions. KCNJ11 was queried in humans (*Homo sapiens*) from May to August 2025.

### 2.1. Searchable Web-Based Programs and Databases Queried

#### 2.1.1. STRING Web-Based Program and Database for Protein–Protein Interactions

STRING is a searchable program to identify and study predicted protein–protein interactions, characterizing genetic mechanisms, molecular functions, and disease pathology with disturbances specific to rare genetic disorders. The interactions may be both direct (physical) and indirect (functional) protein associations, and are derived from genomic context predictions, high-throughput laboratory experiments, and automated text mining from literature sources and other databases with conserved co-expression patterns of genes.

The STRING program provides a statistical approach for analyzing genes and their encoded proteins. It uses four separate criteria including the ***Count in network*** function, which indicates how many proteins are in the visualized network and annotated with a particular term, out of how many proteins in total have this term assigned to them. ***Strength*** describes the size of the enrichment effect as a ratio of the number of term-annotated proteins in a visualized network to the number of proteins expected to be annotated in a random network of the same size (log10(observed/expected)). ***False discovery rate (FDR)*** examines the significance of enrichment with a statistical measurement to conceptualize the rate of type I (false positive) errors in the null hypothesis testing, reported as *p*-values that are corrected for multiple testing within each category. ***Signal*** is defined as a weighted harmonic mean between the observed/expected ratio and −log(FDR). Signal is meant to balance the metrics of larger and smaller terms for more intuitive ordering of enriched terms.

#### 2.1.2. Biological General Repository for Interaction Datasets (BioGRID) for Protein–Protein Interactions

BioGRID (https://theBioGRID.org) is a searchable public database with curated data that is archived for dissemination. It is used for genetic and protein–protein functional interactions from multiple organisms including humans. This searchable website program and its databases allow for the study of related proteins that are assembled with expert input from relevant sources updated monthly and generated on a regular basis for biological interactions. It identifies and focuses on Gene Ontology biological processes, Gene Ontology molecular functions, and Gene Ontology cellular components.

#### 2.1.3. PathwayCommons for Gene–Gene Interactions

PathwayCommons (www.PathwayCommons.org) is another web-based program that uses similar methods to the other two described websites. It focuses on identifying gene regulatory networks and gene–gene functional interactions and pathways. The data are enriched in gene expression and binding information among the interactive or shared processes. These websites provide current curation that focuses on areas of biology to enable insights into conserved networks and pathways relevant to human health and generate validation among shared processes or functions from curated sources involving both gene–gene and protein–protein interaction and associations. They can be utilized to jointly characterize predicted protein–protein associations, functional mechanisms and their protein networks with top biological processes, molecular functions, cellular components, pathways, and disease-gene associations.

## 3. Results

### 3.1. String Protein–Protein Interactions and Functional Analysis

**Figure 2:**
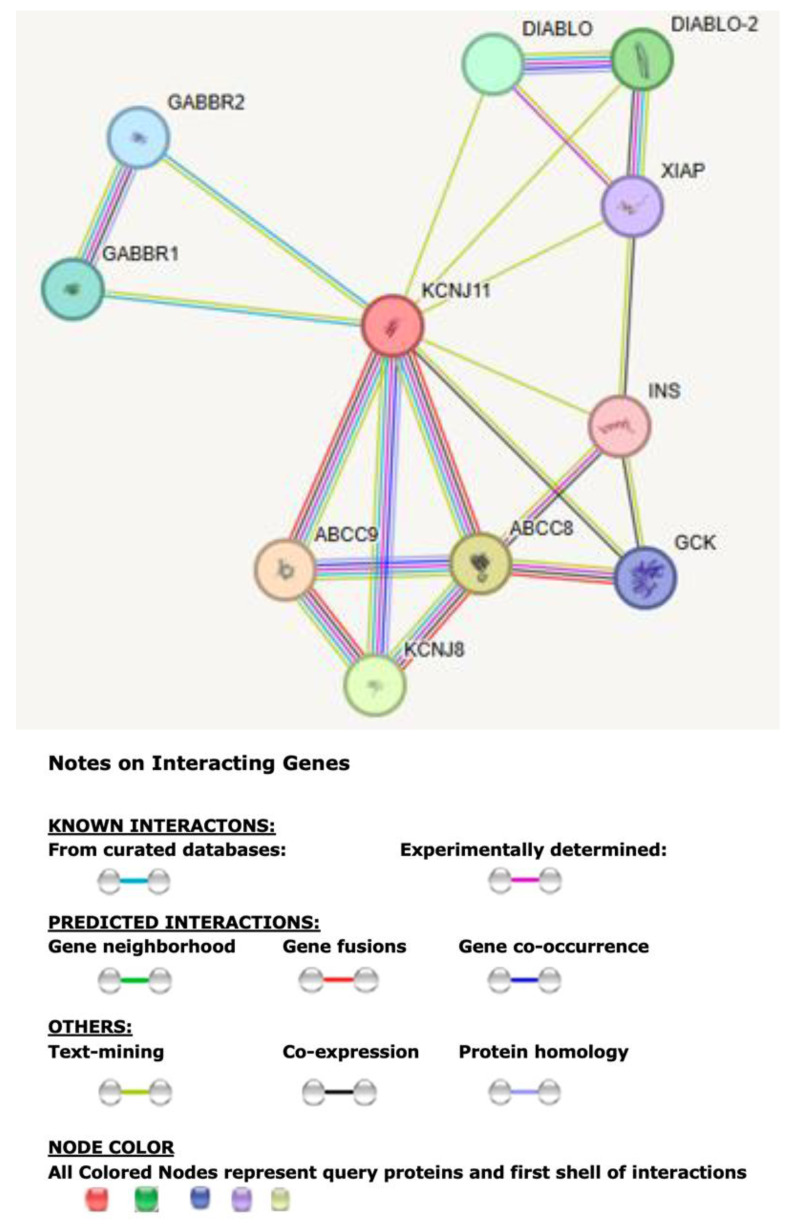
STRING protein–protein first-tier interaction network of *KCNJ11* gene with functional interactions involve 10 associated protein nodes and 21 edges. Edges represent protein–protein associations which are considered specific and meaningful such as proteins that jointly contribute to a shared function.

The searchable STRING analysis for KCNJ11 found 10 interactive protein nodes with each representing all proteins produced including isoforms and 21 edges which indicate both direct and predicted functional and physical protein–protein associations with interactions for each gene along with predicted functional enrichments shown in Figure 2.

The top 10 significantly associated proteins and their genes with predicted functional partners for *KCNJ11* were found and their predicted functions are listed in Table 1. These include *KCNJ8* and ATP-binding cassette sub-family C member (*ABCC*) subunits 8 and 9, whose gene products (Kir6.1, SUR1, and SUR2) form another ATP-sensitive potassium channel (KATP). These combined functions are needed for insulin release. The proteins encoded by *DIABLO* (*605219) or *DIABLO-2* represent the second mitochondria-derived activator of caspases (SMAC), characterized as binding mitochondrial proteins. The *XIAP* gene encodes for the X-linked inhibitor of apoptosis protein (XIAP), a multifunctional E3 ubiquitin protein ligase involved with apoptosis and inflammation, blocking caspases enzymes. Gamma-aminobutyric acid type B receptor 1 (GABBR1) and GABBR2 bind agonists and mediate the coupling of G proteins needed for channel activation and the modulation of downstream effectors. GCK catalyzes the phosphorylation of hexose involved in glucose metabolism and insulin (INS) production. Thus, these cell membrane channels link cellular metabolism to membrane excitability in various cell types including pancreatic beta cells, neurons, endocrine cells, and cardiac and skeletal muscle cells and function to regulate appetite, energy production and conservation, insulin secretion, and the synthesis of fatty acids with beta-oxidation.

The STRING predicted protein functions with the top proteins associated with *KCNJ11* are described in Table 2, including biological processes, molecular functions, cell components, KEGG and Reactome pathways, and disease associations.

### 3.2. BioGRID Protein-Protein Interactions and Analysis

The searchable web-based BioGRID protein–protein functional interaction program and database identified thirteen Gene Ontology Biological Processes; seven Gene Ontology Molecular Functions; and five Gene Ontology Cellular Components associated with KCNJ11. These interactions are listed in Table 3.

### 3.3. PathwayCommons Gene–Gene Interactions and Analysis

**Figure 3:**
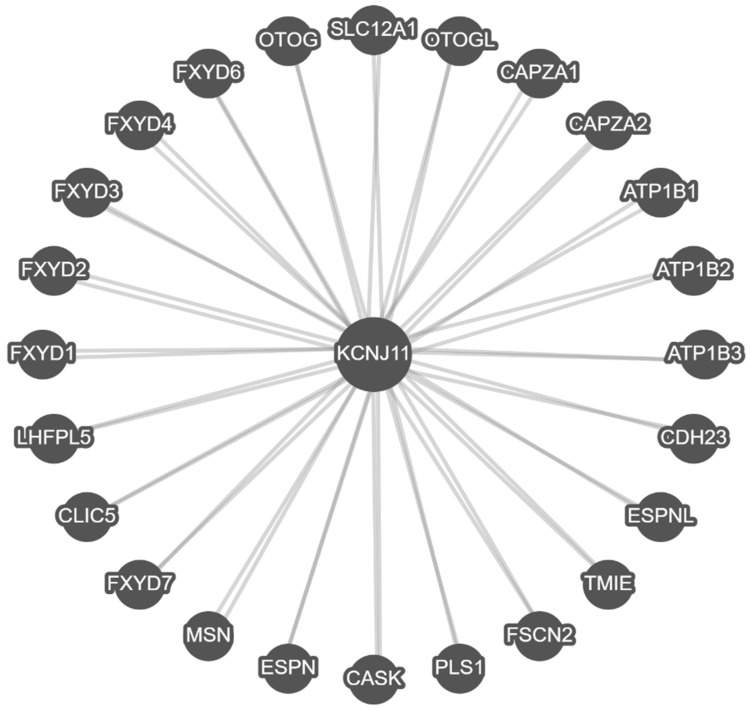
*KCNJ11* gene–gene functional interactions with other related genes.

To study the impact of available gene–gene functional interactions with binding and co-expression data and compare with protein–protein functional interactions, we used a separate searchable web-based program named PathwayCommons (pathwaycommons.org). This program identified 24 interactive genes related to *KCNJ11* as shown in Figure 3.

In Figure 3, *KCNJ11* gene interacts with 24 genes and over-representation of six members of the *FXYD* gene family were found that code for FXYD domain-containing ion transport protein regulators, alternatively named phospholemman. These genes code for small membrane proteins with core motifs and conserved amino acids centered on a single transmembrane span that responds to insulin and adrenergic stimulation (www.omim.org) [64]. Other interactive genes found included *ATP1B1*, *ATP1B2,* and *ATP1B3*, whose encoded proteins are components of plasma membrane pumps for Na^+^/K^+^ ATPases, which have numerous physiological functions to maintain ionic homeostasis critical for cell survival, differentiation, and apoptosis (www.omim.org) [73]. Genes from the *CAPZA* family were involved with the regulation of the growth of actin filament and associated deafness (e.g., *PLS1*, *LHFL5*, *OTOG*, *ESPN*, and *ESPNL*). These relate to transmembrane development and cellular projections. The *MSN* gene is involved with membrane-organizing extension protein assembly. All interactive genes with *KCNJ11* play a role in cell membrane production, structure, and assembly, thereby impacting cell channel development and function, impacting several organ systems (https://apps.pathwaycommons.org/search?type=Pathway&q=KCNJ11, accessed on 15 June 2025).

## 4. Discussion

### 4.1. Background in Prader–Willi Syndrome and Clinical Trials

Prader–Willi syndrome is a classical rare disorder whose variable genetic defects lead to an altered clinical phenotype with hyperphagia and obesity playing a significant role in morbidity and mortality. Diazoxide choline activates the ATP-sensitive potassium (KATP) channel found in pancreatic and other cells and plays a role in insulin release and regulation. In an attempt to predict unanticipated interactions diazoxide choline may have, we analyzed gene–gene, gene–protein, and protein–protein relationships of a key KATP subunit (Kir6.2) and its gene (*KCNJ11*).

### 4.2. Computational Biology and Assessment of KCNJ11

The gene and protein relationships and associations with *KCNJ11* and Kir6.2 uncovered in this in silica investigation loosely grouped into four essential domains of related function. The first is of apoptosis, protein degradation, and inflammation, as seen in the mitochondrial binding proteins (DIABLO-2 and DIABLO), and a multifunctional E3 ubiquitin protein ligase (XIAP). Second, is the coupling of G proteins needed for KATP channel activation, principally GABBR1 and GABBR2, which are GABA type B receptors and are involved with the coupling of G proteins needed for channel activation in pre- and post-synaptic neurons. The third grouping concerns glucose metabolism and control, including insulin production and release. Six members of the *FXYD* gene family (*FXYD1*, *2*, *3*, *4*, *6*, and *7*) share gene interactions with *KCNJ11*, and code for ion regulators or small membrane proteins that play a role in insulin release and adrenergic stimulation (www.omim.org). The *GCK* gene product catalyzes the phosphorylation of hexose, a key factor in glucose metabolism in the liver and pancreas, as well as insulin production. The fourth domain of related functions has to do with the maintenance of intracellular ionic homeostasis. The proteins thus associated with *KCNJ11* include the ATP-binding cassette members (e.g., *ABCC8*, *ABCC9,* and *KCNJ8*) which form the channel pore, and whose function is needed for insulin release and regulation. In addition, gene–gene interactions were found between *KCNJ11* and members of the *ATP1B1–3* family, which encode subunits for Na^+^/K^+^ ATPase. These transporters are critical for maintaining intracellular ionic homeostasis, cellular excitability, and metabolic regulation.

Many of the above genes and their encoded proteins have strongly interrelated functions, and could arguably be placed in multiple functional domains. What is important is the theoretical vulnerability to functional disruption in the presence of diazoxide choline. For example, those with PWS deletion type 1 lack *NIPA1* and *NIPA2,* genes for cation transporter proteins, and could experience electrolyte stability, especially of potassium and indirectly of magnesium while undergoing diazoxide treatment [9,15,18]. Pipatpolkai et al. [74] reported on the potential impact of altered cation transport on the KATP as it related to neonatal diabetes mellitus. The interplay between ATP and ADP, which close the channel, and magnesium-ATP (MgATP) and MgADP, which increase channel activity, is integral to KATP function [74]. But diet control and restrictions could affect the bioavailability and reserves of magnesium and calcium cations in those with PWS, exacerbated by lower muscle and bone mass, resulting in lowered magnesium levels [18]. Similarly, the lack of the *MAGEL2* gene could affect patients with PWS receiving diazoxide choline treatment. The gene, of the MAGE family of ubiquitin ligase regulators, is maternally imprinted, paternally expressed in the 15q11–q13 region [4]. It is fundamental to the process of recycling cell membrane proteins and neuropeptide production in the hypothalamus [75]. Whether these genetically abnormal processes could alter KATP channel activation when targeted by diazoxide choline is unknown. Together, these interactions could underscore the need for genotype-informed monitoring protocols.

Many of the identified genes play a role in cell membrane production and structure in several organ systems. In the cells of the pancreas, the KATP channels link cellular metabolism to membrane excitability, regulating energy production and storage, appetite regulation, insulin production, and fatty acid synthesis and oxidation. These functions are all key in treating hyperphagia and obesity in PWS. But the ubiquitousness of KATP channels suggests that diazoxide choline may potentially influence neuronal, endocrine, cardiac, and skeletal muscle cells, and theoretically initiate or exacerbate common findings associated with PWS, such as edema, hypertrichosis, skeletal muscle hypotonia, cardiac dysfunction, and neuro-glial cell signaling that impacts brain and neurological tissue. Existing metabolic disorders, and disturbed bone metabolism and mineral storage could be additional risk factors, but more studies are needed.

### 4.3. Study Limitations

The central limitation of our study is its theoretical nature. The rarity of a rare disease makes designing and executing a clinical trial with a patient base large enough to adequately represent the variable genetic defects, phenotypic heterogeneity, and disturbed biology extremely challenging, if not prohibitively expensive [3,24,76]. The ability to determine the potential risks portfolio of a new medication currently depends on such a large subject base. In an effort to fill the knowledge gap, we used the in silico approach of computational biology to identify possible unanticipated adverse effects of diazoxide choline on persons with PWS. We are not able to verify these findings in situ, and practically speaking, only time will tell. But this approach provides practitioners guidance as to potential inadvertent effects for which to monitor a patient with PWS taking diazoxide choline.

### 4.4. Future Studies in Prader–Willi Syndrome

There are hundreds of genes in the 15q11–q13 Prader–Willi syndrome critical region that could potentially have epigenetic effects outside the region, with interpatient diversity. To both understand an individual’s specific gene abnormalities within the PWS critical region, and possible interactions outside the region, whole DNA genome or exome sequencing could be considered. This could lead to comprehensive individualized pharmacogenetics for medication management and treatment, similar to the treatment of cystic fibrosis (CF). Although the condition is caused by mutations in the single gene encoding the cystic fibrosis transmembrane conductance regulator (CFTR) protein, phenotypic heterogeneity occurs related to the type of mutation, whether it causes a partial or complete absence of the protein, a malformed protein, or simply one that does not appropriately function. The concept of theratyping, matching specific drugs to particular mutations, provides the potential to assess a patient’s response to current and emerging therapies [77,78,79]. This allows clinical trials for those with CF having the Phe508del *CFTR* gene defect only [80].

## 5. Conclusions

Diazoxide choline’s mechanism of action is to activate KATP, indirectly inhibiting the release of insulin. By uncovering the functional protein and gene associations of a subunit of KATP, we found a wide range of related functions, including autophagy, the coupling of G proteins needed for KATP channel activation, factors in glucose metabolism and control, and the maintenance of intracellular ionic homeostasis. These findings suggest that diazoxide choline may influence several biological systems beyond pancreatic and metabolic functions. As such, altering those processes could lead to rare and as yet unidentified complications. Further analysis and study may lead to more comprehensive guidelines on monitoring for adverse reactions for patients with PWS treated with diazoxide choline.

Rare diseases, such as PWS, inherently present clinical barriers and limitations for treatment discovery, due to genetic heterogeneity and a small recruitable patient pool for clinical studies. In the future, in-depth condition-specific as well as individualized pharmacogenetics may impact the selection and success of a drug, as an important adjunct to clinical drug trials.

## Figures and Tables

**Table 1:** STRING: Protein or gene symbols and descriptions of the top ten proteins associated with *KCNJ11* *.

Protein and/or Gene Symbol	Description
***ABCC9***	ATP-binding cassette sub-family C member 9, a subunit of ATP-sensitive potassium channel (KATP) that forms the channel pore belonging to the ABC transporter superfamily. Encodes sulfonylurea receptor 2 (SUR2).
***ABCC8***	ATP-binding cassette sub-family C member 8, a subunit of beta-cell ATP-sensitive potassium channel (KATP) that forms the channel pore belonging to the ABC transporter superfamily and acts as a regulator for insulin release. Encodes sulfonylurea receptor 1 (SUR1).
***KCNJ8***	ATP-sensitive inward rectifier potassium channel 8 controlled by G proteins regulating potassium flow with inward rectification mainly due to the blockage of outward current by internal magnesium. Encodes the transmembrane potassium inward-rectifying Kir6.1.
***DIABLO* or *DIABLO-2***	Diablo IAP-binding mitochondrial protein promotes apoptosis by activating caspases in the cytochrome c/Apaf-1/caspase-9 pathway by opposing the inhibitory activity of the inhibitor of apoptosis proteins (IAP).
***GABBR1***	Gamma-aminobutyric acid type B receptor subunit 1 as a component of a heterodimeric G protein-coupled receptor for GABA, formed by GABBR1 and GABBR2, but GABBR1 only binds agonists. Ligand binding causes a conformational change that triggers signaling via guanine nucleotide-binding proteins (G proteins) and modulates the activity of downstream effectors such as adenylate cyclase.
***GABBR2***	Gamma-aminobutyric acid type B receptor subunit 2 as a component of a heterodimeric G protein-coupled receptor for GABA, formed by GABBR1 and GABBR2, but GABBR1 only binds agonists while GABBR2 mediates coupling to G proteins. Ligand binding causes a conformational change that triggers signaling via guanine nucleotide-binding proteins (G proteins) and modulates the activity of downstream effectors such as adenylate cyclase.
***GCK***	Hexokinase-4, or glucokinase, catalyzes the phosphorylation of hexose, such as D-glucose, D-fructose, and D-mannose to hexose 6-phosphate and D-mannose 6-phosphate, respectively, and involves the first step in glycolysis.
***XIAP***	E3 ubiquitin-protein ligase, a multifunctional protein which regulates not only caspases and apoptosis but also modulates inflammatory signaling and immunity, copper homeostasis, mitogenic kinase signaling, cell proliferation and invasion, and metastasis. It acts as a direct caspase inhibitor and targets proteins for degradation.
***INS***	Insulin A chain which decreases blood glucose concentration and increases cell permeability to monosaccharides, amino acids, and fatty acids with the acceleration of glycolysis and glycogen synthesis in the liver.

* STRING database (www.string-db.org); GeneCards database (www.genecards.org). The ten protein nodes are arranged by their predicted functional analytical order with genes *ABCC9* and *ABCC8* representing ATP-binding cassettes tied for the highest-ranking score at the top of the list of encoded proteins; these are key to carrying out the function of *KCNJ11*.

**Table 2:** STRING: Predicted functions for KCNJ11 *.

Biological Process	CIN ^A^	Strength ^B^	Signal ^C^	FDR ^D^
Neuron-glial cell signaling (GO:0150099)	2 of 5	2.86	1.25	0.0054
Potassium ion import across plasma membrane (GO:1990573)	4 of 44	2.21	1.97	0.00016
Negative regulation of protein secretion (GO:0050709)	3 of 62	1.94	1.17	0.0054
Negative regulation of secretion by cell (GO:1903531)	4 of 141	1.71	1.32	0.0016
Regulation of peptide hormone secretion (GO:0090276)	5 of 185	1.68	1.60	0.00027
**Molecular Function**	**CIN**	**Strength**	**Signal**	**FDR**
Sulfonylurea receptor activity (GO:0008281)	2 of 2	3.25	1.60	0.0013
G protein-coupled GABA receptor activity (GO:0004965)	2 of 3	3.08	1.55	0.0016
ATP-activated inward rectifier potassium channel activity (GO:0015272)	3 of 5	3.03	2.45	3.57 × 10^−5^
ATPase-coupled cation transmembrane transporter activity (GO:0019829)	4 of 53	2.13	2.17	5.04 × 10^−5^
Potassium channel activity (GO:0005267)	4 of 126	1.75	1.52	0.00058
**Cellular Component**	**CIN**	**Strength**	**Signal**	**FDR**
Inward rectifying potassium channel (GO:0008282)	4 of 4	3.25	4.47	7.51 × 10^−9^
Potassium ion-transporting ATPase complex (GO:0031004)	3 of 3	3.25	3.18	1.76 × 10^−6^
G protein-coupled GABA receptor complex (GO:1902712)	2 of 2	3.25	1.92	0.00035
G protein-coupled receptor heterodimeric complex (GO:0038039)	2 of 3	3.08	1.81	0.00053
Plasma membrane protein complex (GO:0098797)	8 of 589	1.39	2.01	1.05 × 10^−7^
**KEGG Pathway**	**CIN**	**Strength**	**Signal**	**FDR**
Type II diabetes mellitus (hsa04930)	4 of 45	2.20	2.73	3.69 × 10^−6^
Maturity onset diabetes of the young (hsa04950)	2 of 25	2.16	1.11	0.0083
Insulin secretion (hsa04911)	4 of 82	1.94	2.25	1.83 × 10^−5^
GnRH secretion (hsa04929)	3 of 63	1.93	1.58	0.00065
Apoptosis—multiple species (hsa04215)	2 of 30	2.08	1.07	0.0094
**Reactome Pathway**	**CIN**	**Strength**	**Signal**	**FDR**
ATP-sensitive potassium channels (HSA-1296025)	4 of 4	3.25	4.61	4.19 × 10^−9^
Inwardly rectifying K+ channels (HSA-1296065)	6 of 35	2.49	5.15	5.78 × 10^−11^
Defective ABCC8 can cause hypo- and hyperglycemias (HSA-5683177)	2 of 2	3.25	1.72	0.00078
Defective ABCC9 causes CMD10, ATFB12, and Cantu syndrome (HSA-5678420)	2 of 2	3.25	1.72	0.00078
Disorders of transmembrane transporters (HSA-5619115)	4 of 176	1.61	1.39	0.00078
**Disease-Gene Association**	**CIN**	**Strength**	**Signal**	**FDR**
Hypertrichotic osteochondrodysplasia Cantu type (DOID:0060569)	3 of 3	3.25	3.11	2.37 × 10^−6^
Permanent neonatal diabetes mellitus (DOID:0060639)	4 of 6	3.08	3.99	5.06 × 10^−8^
Gestational diabetes (DOID:11714)	3 of 9	2.78	2.54	2.17 × 10^−5^
Maturity-onset diabetes of the young (DOID:0050524)	4 of 14	2.71	3.54	2.45 × 10^−7^
Hypoglycemia (DOID:9993)	4 of 22	2.51	3.18	8.96 × 10^−7^

* STRING predicted functions for KCNJ11 with biological processes, molecular functions, cellular components, pathways, and disease-gene associations. ^A^ CIN (count-in-network) indicates how many proteins in the network are annotated with a particular term and how many proteins in total (in the network and in the background) have this term assigned to this variable per category (Biological Process, Molecular Function, etc.). ^B^ Log10(observed/expected) or strength. This measure describes how large the enrichment effect is with the ratio between (i) the number of proteins in the network that are annotated with a term and (ii) the number of proteins expected to be annotated with this term in a random network of the same size. ^C^ Signal is defined as a weighted harmonic mean between the observed/expected ratio and −log(FDR). FDR or false discovery rate tends to emphasize larger terms due to their potential for achieving lower *p*-values, while the observed/expected ratio highlights smaller terms, which have a high foreground to background ratio but cannot achieve low FDR values due to their size. ^D^ FDR is a statistical measure that describes the significance of enrichment. Shown are *p*-values corrected for multiple testing within each category.

**Table 3:** Gene Ontology Biological Processes, Molecular Functions and Cellular Components using BioGRID protein–protein functional interaction for KCNJ11.

Biological Processes	Molecular Functions	Cellular Components
Energy reserve metabolic process	ATP binding	ATP-sensitive potassium channel complex
Glucose metabolic process	ATP-activated inward rectifier potassium channel activity	Muscle T-tubule
Negative regulation of insulin secretion	G protein-activated inward rectifier potassium channel activity	Integral component of plasma membrane
Neurological system process	Ankyrin binding	Plasma membrane
Potassium ion import	Ion channel binding	Voltage-gated potassium channel complex
Potassium ion transmembrane transport	Potassium ion binding	
Regulation of membrane potential	Voltage-gated potassium channel activity	
Response to ATP		
Response to drug		
Small molecule metabolic process		
Synaptic transmission		
Regulation of insulin secretion		
Regulation of ion transmembrane transport		

## Data Availability

The original contributions presented in this study are included in the article. Further inquiries can be directed to the corresponding author.
